# Contrast-enhanced abdominal CT compared with splenoportography in postliver transplant with giant varix and thrombus in portal vein: Case report

**DOI:** 10.1016/j.radcr.2024.09.018

**Published:** 2024-09-24

**Authors:** Jacub Pandelaki, Prijo Sidipratomo, Hanifah Oswari, Sastiono, Sahat Basana Romanti Ezer Matondang, Krishna Pandu Wicaksono, Heltara Ramandika, Yuzana Tiarasia, Kukuh Nurcahyo, Jason, Gideon Hot Partogi Sinaga

**Affiliations:** a Department of Radiology, Dr. Cipto Mangunkusumo National General Hospital, Faculty of Medicine, Universitas Indonesia, Jakarta, Indonesia; b Department of Pediatric, Dr. Cipto Mangunkusumo National General Hospital, Faculty of Medicine, Universitas Indonesia, Jakarta, Indonesia; c Department of Pediatric Surgery, Dr. Cipto Mangunkusumo National General Hospital, Faculty of Medicine, Universitas Indonesia, Jakarta, Indonesia; d Faculty of Medicine, Universitas Indonesia, Jakarta, Indonesia

**Keywords:** Contrast-enhanced CT, Splenoportography, Varix of the portal vein, Portal vein thrombus, Liver transplant

## Abstract

Imaging remains an essential aspect in evaluating patients receiving liver transplants, especially in cases of complications such as portal vein thrombosis. Several imaging modalities are available to approach portal vein thrombosis, with portography as the gold standard. However, the development of noninvasive methods such as contrast-enhanced computed tomography (CECT) is preferred nowadays due to the fewer complications in nature. This case report presented a case of a giant varix of the portal vein with thrombus in a 20-year-old male receiving living-donor liver transplant, reliably visualized in both CECT and direct splenoportography. Detailed parameters and sequences required for accurate imaging in CECT are discussed in this study.

## Introduction

Vascular examinations and interventions directly involving the portal vein should be done cautiously due to the high risk of complications [1]. In the cases of liver transplant, including living-donor liver transplant (LDLT), postoperative imaging is essential due to the risk of several complications, such as portal hypertension and thrombus formation [2]. Portography, a conventional minimally-invasive visualization of the portal vein, remains the gold standard due to the ability to identify the portal vein morphology and to measure the portal venous pressure [3]. However, although the evidence is still scarce, noninvasive imaging such as contrast-enhanced computed tomography (CECT) might be sufficient to evaluate liver transplant complications [4]. This case report presented a comparison between CECT and splenoportography, both indirect and direct, in assessing post-LDLT recipient with a thrombus inside a giant varix at the main portal vein.

## Case presentation

A 20-year-old male was admitted to our emergency department for losing consciousness. The patient was diagnosed with biliary atresia at 3 weeks old and was initially planned for bypass surgery. However, the procedure was canceled due to liver cirrhosis, and the patient underwent a liver transplant in Japan at 6 months old. For the next 20 years, the patient experienced no significant health issues. Three years prior to the current visit, the patient began experiencing malaise which persisted for 2 years. There was a history of recurrent upper gastrointestinal bleeding in the last 4 months, while clinical examination showed ascites with infection. Blood test revealed an increase in indirect, direct, and total bilirubin levels to 7.16, 17.13, and 24.3 mg/dL, respectively. After resolving the emergencies, an endoscopic examination showed esophageal varices without active bleeding. The patient was then referred to the radiology department with suspicion of increased portal venous pressure.

**Fig. 1:**
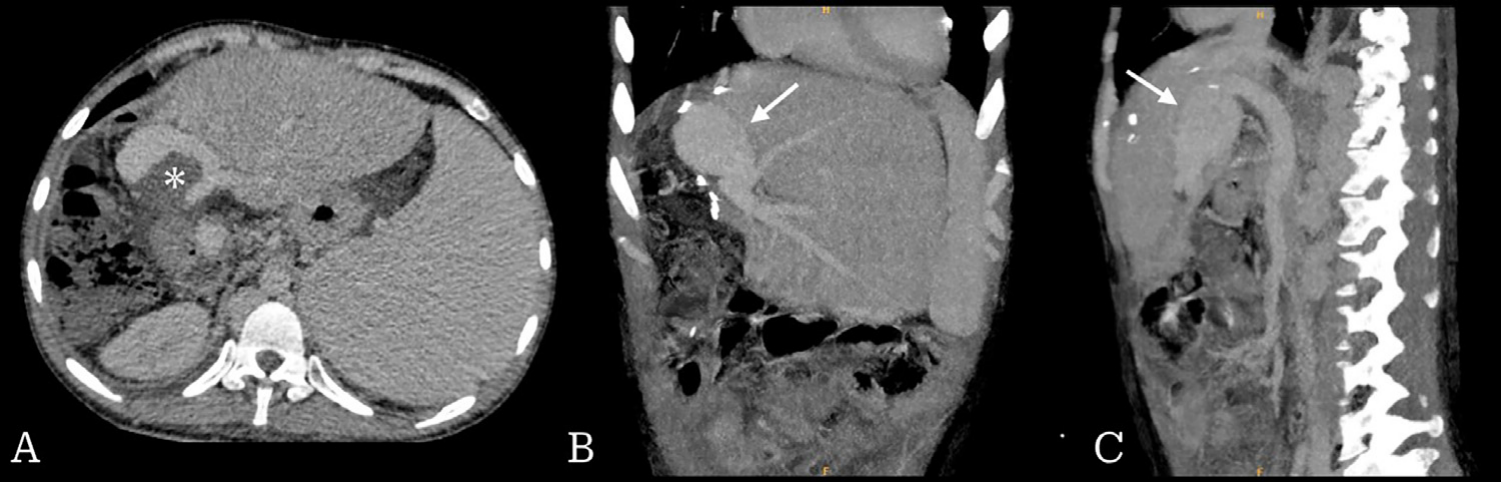
Contrast-enhanced abdominal CT: (A) Axial view showed thrombus (asterisk) in the main portal vein. (B) Coronal and (C) sagittal view showed varix in the main portal vein (arrow).

An abdominal CECT scan was conducted through a single acquisition protocol with subsequent related parameters (Table 1). Monophasic contrast was performed with 80 mL iodine-based contrast and 40 mL saline, injected with a 2 mL/kg dose. CT scan was set with chaser mode at 3 mL/s, and the acquisition of the portal vein was set to 50 seconds after the contrast injection. The abdominal CECT showed a giant varix of the extrahepatic portal vein, with an incidental visualization of intraluminal thrombus in the hilus region (Fig. 1). Due to the absence of clinical improvement after pharmacological treatments, the patient was referred to the interventional radiology division for splenoportography to confirm the diagnosis and planned for the thrombectomy procedure to extract the thrombus and improve blood flow. Initially, invasive splenoportography would have been preferred for its ability to measure pressures. However, due to the lack of coverage by insurance and the unavailability of necessary equipment, only visualization was performed.

**Table 1:** Contrast-enhanced CT scan abdomen parameter in Cipto Mangunkusumo hospital.

Abdominal CECT parameter	Noncontrast	Contrast
kV	120	120
mAs	142	142
Scan time	7.3 s	7.3 s
Postinjection delay	-	50-70 s
Pitch	0.800	0.800
Rotation	0.5	0.5
Liver area dose right index	+3	+3

**Fig. 2:**
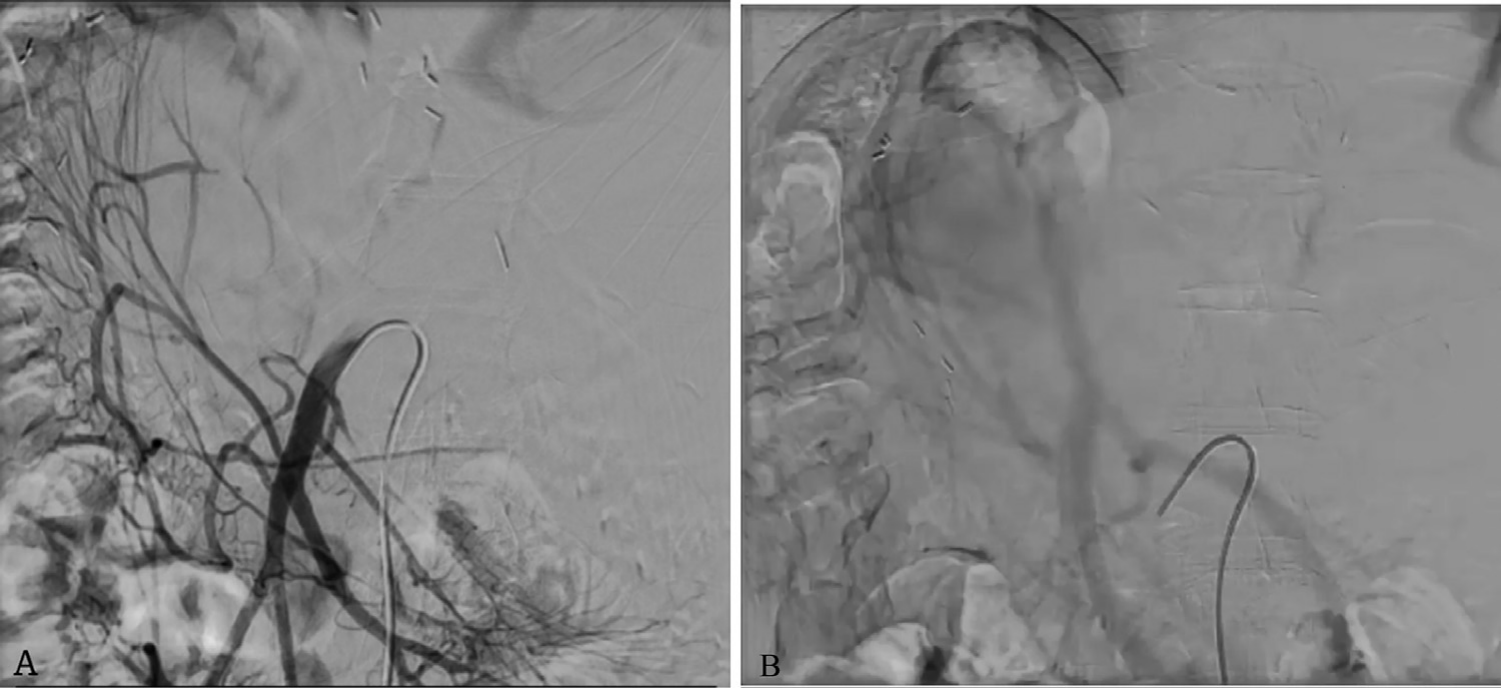
Indirect splenoportography via superior mesenteric artery in (A) arterial and (B) venous phase showed unclear visualization of the varix and thrombus.

**Fig. 3:**
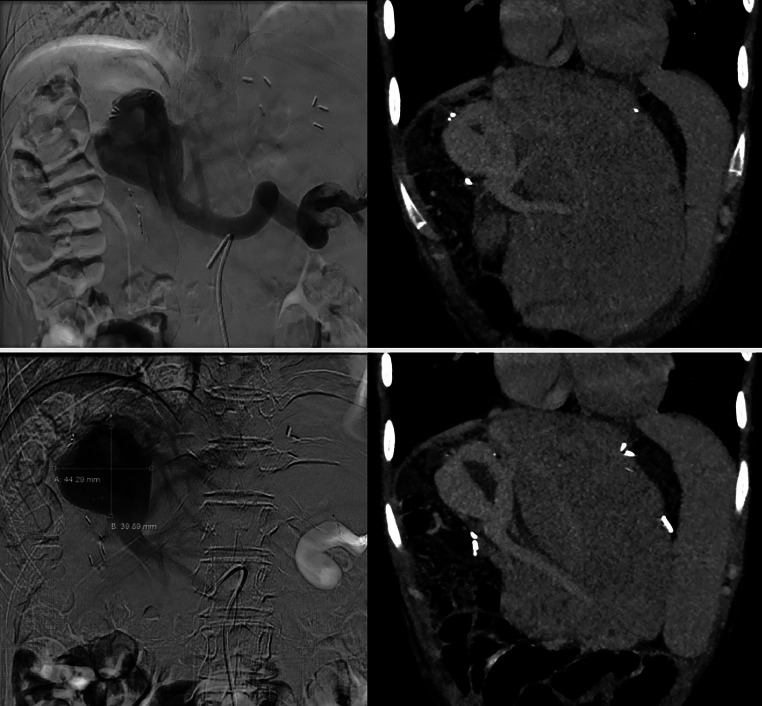
Side-to-side comparison between the direct splenoportography (left) and CECT (right), showing varix with estimated size of 44.3 × 39.9 mm and an intraluminal thrombus.

Indirect splenoportography was performed under local anesthesia. Vascular access was made in the right femoral artery through ultrasound guidance with contrast evaluation from the superior mesenteric artery. However, the indirect splenoportography failed to visualize the varix or the thrombus (Fig. 2). Hence, the procedure was continued with a direct splenoportography to confirm the diagnosis. Direct splenoportography was performed under general anesthesia. Skin puncture was made with an 18G IV cannula under ultrasound guidance. The contrast was directly injected into the spleen intraparenchymal perihilar vein. The digital subtraction angiography (DSA) technique confirmed the giant varix of the portal vein, with a dimension of approximately 44.29 × 39.89 mm, and the intraluminal thrombus (Fig. 3). The patient was referred to the digestive surgery division for further treatment. Unfortunately, the patient passed away 3 months later due to septic peritonitis.

## Discussion

Although rarely present in long-term complications, portal venous outflow complications, such as PVT, might occur as one of the complications after receiving LDLT [5]. PVT has an incidence of around 3%-7%, with clinical manifestations mostly the same as portal hypertension due to the extrahepatic blockage [5,6]. Decreased portal venous blood supply may lead to liver failure, while portal hypertension leads to splenomegaly, esophageal varices and bleeding, intestinal edema, and ascites [7]. In this case, an extensive diagnostic workout in the form of endoscopy for the esophageal varices and advanced imaging modalities to find the primary cause is deemed necessary to devise proper management [2,^4^,8].

Several imaging modalities are available to approach portal vein thrombosis in liver transplant recipients. Color-doppler or contrast-enhanced ultrasound is practical in the initial diagnosis and capable of evaluating vascular hemodynamics. However, ultrasound is heavily operator-dependent and has limited evidence of capabilities in evaluating postliver transplants. Both computed tomography (CT) and magnetic resonance (MR) provide a wider field of view, thus making them more sensitive in detecting a broader spectrum of PVT features. Portography is the gold standard in visualizing PVT with high resolution and giving options for portal vein pressure measurement and catheter-based management [9]. Nevertheless, our study presented a more satisfactory result from the tailored dynamic CECT than the direct splenoportography.

**Fig. 4:**
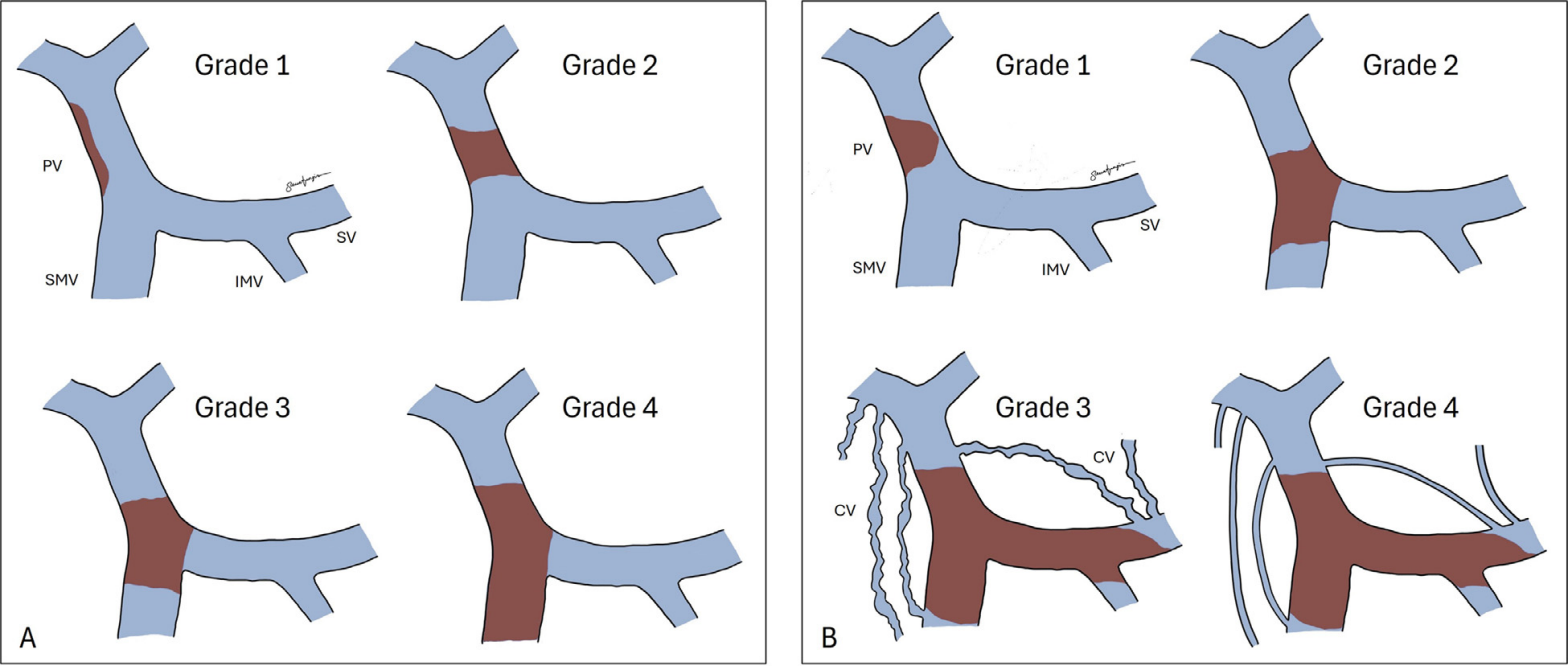
Classification of PVT. (A) Classification by Yerdel et al.: Grade 1: <50% PVT, with or without minimal extension into the SMV; Grade 2: 50-100% PVT, with or without minimal extension into the SMV; Grade 3: Complete thrombosis of both PV and proximal SMV, but with patent distal SMV; Grade 4: Complete thrombosis of PV, proximal SMV, and distal SMV. (B) Classification by Charco et al.: Grade 1: Thrombosis limited to portal vein; Grade 2: Thrombosis extending to proximal SMV; Grade 3: Diffuse thrombosis of the splanchnic system with dilated collateral veins; Grade 4: Diffuse thrombosis of the splanchnic system with fine collateral veins. CV, Collateral vein; IMV, Inferior mesenteric vein; PV, Portal vein, SV, Splenic vein, SMV, Superior mesenteric vein.

Several grading systems of PVT are available, with classification from Yerdel et al. being the most widely used, while another classification by Charco et al. helps reinforce the planning of interventional management (Fig. 4) [10,11]. In our case, both CECT and direct splenoportography confirmed the presence of thrombus locally in the dilated portal vein without any extension to the splanchnic system, suggesting an example of grade 1 PVT in both Yerdel et al. and Charco et al. classification. Although direct splenoportography could give the best visualization of collateral veins, leakage, or stenosis in the splanchnic system, the absence is confirmed in both our CT scan and direct splenoportography examination.

The European Association for the Study of Liver Diseases (EASL) recommends a triple-phase liver CT protocol for evaluating portal vein obstruction. For contrast injection, typically 1.5 mL/kg (350 mOsm/L) or 2.0 mL/kg (300 mOsm/L) up to 150 mL of nonionic contrast is injected at a rate of 3 to 5 mL/s, where a higher flow rate results in better enhancement. The late arterial phase begins 40 seconds after contrast injection, the portal venous phase occurs 20 seconds after the arterial phase or 75 seconds after contrast injection, and the delayed phase is conducted 7 to 10 min after the portal phase. All scans are performed during a breath-hold on inspiration to minimize motion artifacts [12]. In our protocol, the regular abdominal CT contrast-enhanced parameter has a scanning time of 10-25 seconds faster, with contrast given at a slower rate (2.5-3 mL/s) compared to the venous phase.

## Conclusion

This study compared CECT and splenoportography, in which both effectively visualize the thrombosis in the varix of the portal vein in post-LDLT patient. Although splenoportography still stands as the gold standard for detecting abnormalities in the portal vasculature, a contrast-enhanced abdominal CT scan with a standardized protocol in our center could produce a comparable imaging quality to splenoportography.

## Patient consent

The authors have obtained consent from the patient for their data, including their medical history and imaging studies, to be published in this case report.
